# Spatial-temporal modeling of background radiation using mobile sensor networks

**DOI:** 10.1371/journal.pone.0205092

**Published:** 2018-10-19

**Authors:** Zheng Liu, Shiva Abbaszadeh, Clair Julia Sullivan

**Affiliations:** Department of Nuclear, Plasma, and Radiological Engineering, University of Illinois at Urbana-Champaign, Urbana, IL, United States of America; University of Iowa, UNITED STATES

## Abstract

Modeling of background radiation for the urban environment plays an important role in homeland security. However, background radiation is difficult to assess due to its spatial-temporal fluctuations caused by the variation in soil composition, building materials, and weather patterns *etc*. To address the challenge of background radiation modeling, we developed a mobile sensor network to continuously monitor the background radiation; we also proposed a maximum likelihood estimation algorithm to decouple and estimate the background’s spatial distribution and temporal fluctuation. Experimental results demonstrated how this background radiation monitoring system accurately recognized high background regions in the experimental area, and successfully captured temporal fluctuation trends of background radiation during rains. Our system provides an efficient solution to model the temporal fluctuation and spatial distribution of background radiation.

## Introduction

In the area of homeland security, environmental monitoring, and radiation regulation, sensor networks have been used to monitor a geographic region’s radiation level and detect anomalous radiation sources [1–3]. Different algorithms have been developed to estimate the locations and intensities of anomalous radiation sources [4–9]. However, most of these methods require prior knowledge of background radiation that is usually unavailable. Thus they make assumptions about the background radiation. Further, these methods are designed for one-time experiments that do not take advantage of historical measurements. As a result, applying sensor networks for long-time radiation observations and modeling background radiation for areas of interest has been a critical missing step in radiation detection with sensor networks. To address this important challenge, we built a mobile sensor network with a data streaming/storage system for long-time radiation observations. We also proposed a background radiation estimation algorithm (named the BR-MLE algorithm) that models the spatial distribution and temporal fluctuation of background radiation based on measurements from the mobile sensor network.

The knowledge of background radiation plays an important role in the anomalous source detection. There are two major approaches that are commonly used to detect anomalous radiation sources using data from sensor networks: the maximum likelihood estimation-based methods (MLE methods) [4–6, 10], and the Bayesian estimation-based methods (Bayesian methods) [7–9]. Both of these two methods require the prior knowledge of background radiation. For the MLE methods, most of the algorithms assume that the background radiation is known and uniformly distributed in the experimental area [4–6]. However, the background radiation is usually non-uniformly distributed due to the naturally-occurring radioactive materials (NORMs) presented in air, soil and building materials. High background areas caused by NORMs may be mistakenly identified as anomalous sources, and this introduces false alarms in the anomalous source detection. In [10], the spatial distribution of background radiation was taken into account during the source detection experiment. When initializing their MLE algorithm, they manually divided the experimental area into high and low background regions. This required the prior knowledge about background radiation of their experimental area. In Bayesian methods, the prior contains the original knowledge about background radiation and sources. During an experiment, this knowledge is updated on new measurements, and source parameters are estimated through the posterior distribution. The performance of the Bayesian methods heavily depends on the prior knowledge, and most of the Bayesian algorithms assume background radiation is known [7–9].

Although the knowledge of background radiation is vital in the anomalous source detection, the background radiation is not trivial to model because it always fluctuates in space and time. There are three major decay chains presented in the terrestrial background radiation, thorium (^232^Th), uranium (^238^U), and potassium (^40^K) [11]. The spatial distribution of these radioactive isotopes in the environment leads to the spatial distribution of background radiation. For example, a marble square will create a high background region in a city. The temporal fluctuation of background radiation is caused by a variety of reasons, especially the precipitation. It has long been observed that precipitation can induce an elevation of background radiation [12]. During a rainfall, the scavenging effect of rain and snow brings radioactive materials in the upper air down to the ground and elevates the background radiation. ^214^Pb and ^214^Bi are the major contributors to the elevated background radiation, and thus the radiation fluctuation peaks usually have a duration of several hours according to those isotope’s half life [13, 14].

The major contribution of this paper is the development of a real-time data streaming mobile sensor network and a maximum-likelihood based algorithm for background radiation modeling (the BR-MLE algorithm). This provides an efficient solution to long-term monitoring of an area’s radiation and to model the detailed background radiation distribution in both space and time. In this paper, we first introduce the mobile sensor network system, including the hardware components and the data streaming pipe-line. Then, we present the BR-MLE algorithm for background radiation estimation. This algorithm utilizes long-term radiation measurements from sensor networks to estimate background radiation. To demonstrate this algorithm, we deployed a one-node sensor network on the campus of University of Illinois, and applied the BR-MLE algorithm to estimate the spatial distribution and temporal fluctuation of background radiation.

## Materials and methods

### Mobile sensor network

**Fig 1:**
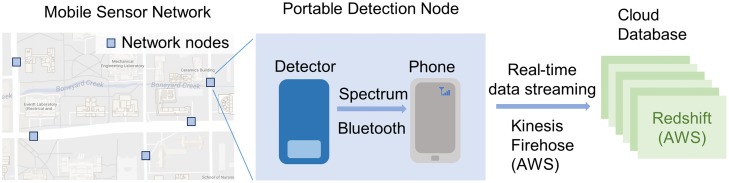
Configuration of the mobile sensor network and its data streaming/storage system. This mobile sensor network has identical detection nodes. In each of these detection nodes, there is a radiation detector (D3S detector) connected with a phone through Bluetooth. An Android application was developed to control the data transmitting pipe-line. In each second, the detector acquires a spectrum and sends it to the phone. The phone then streams this spectrum, the node’s current GPS location, and the current time stamp to a cloud database. This data streaming system was built upon the Amazon Kinesis Firehose, and the cloud database was built upon the Amazon Redshift database.

A mobile radiation sensor network was designed and built to monitor the background radiation of areas of interest, such as the campus of University of Illinois. As shown in Fig 1, this sensor network contains several identical nodes, and each node is composed of an integrated gamma-ray and thermal neutron detector (the D3S detector) [15] and a smart phone (Samsung Galaxy S6 with Android 6.0.1). In this study, we focused on the gamma-ray radiation and thus only used the gamma-ray detector inside the D3S detector. This gamma-ray detector is a thallium activated cesium iodide (CsI(Tl)) scintillation detector with silicon photo-multiplier as the readout system. The detector’s crystal size is 2 × 1 × 0.5 inch. For applications in the security area, this detector is pre-calibrated by Kromek to have a gamma-ray detection range between 30keV to 3MeV, an energy resolution of 7% at 662 keV, and a maximum throughput of 10000 counts per second (cps) for gamma channel. The smart phone acts as a node computer to control the D3S detector, provide GPS information, and store/stream measurements. The smart phone controls the detector and receives radiation measurements through Bluetooth serial interfaces. The data streaming pipe-line was developed upon the platform of Amazon Web Service (AWS). A Kinesis Firehose data streaming system was setup to stream data from all the detection nodes of the mobile sensor network to a cloud database. This cloud database was built using the AWS Redshift database service. In a general usage scenario, the detector acquires a spectrum and sends this spectrum to the phone via Bluetooth. The phone then streams this spectrum as well as the current GPS location and time stamp to the Redshift database through Kinesis Firehose. Both the hardware and the data streaming/storage system are easily scalable. For the whole system, the maximum latency is less than 300 seconds.

### Background radiation—Maximum likelihood estimation algorithm (BR-MLE)

A background radiation maximum likelihood estimation (BR-MLE) algorithm was developed to model the background radiation using measurements from mobile sensor networks. Although our mobile sensor network is capable of measuring gamma-ray spectra, we focused on the gross count rate of background radiation as an initial study. This algorithm estimates the spatial distribution and temporal fluctuation of background radiation by constructing a series of Poisson models for background radiation and estimating the Poisson means for different locations at different times through maximum likelihood estimation framework.

Suppose the mobile sensor network collects a dataset D containing *N* measurements, which is denoted by $D=\{d_{1},d_{2},\ldots ,d_{N}\}$. According to the nature of radiation emission, those measurements can be modeled by a series of Poisson distributions. The *n*’th measurement *d*_*n*_ can be treated as a random sample from Poisson distribution *P*_*n*_ with mean value λ_*n*_. λ_*n*_ indicates the background radiation level when the measurement *d*_*n*_ is acquired. Since the mobile sensor network collects measurements from different locations at different times, and the background radiation level has spatial and temporal distributions, λ_*n*_ may be different from each other. Those λ_*n*_ can be summarized by a single function λ(*x*, *y*, *t*) whose independent arguments are location and time. If the *n*′*th* measurement *d*_*n*_ is taken at location (*x*_*n*_, *y*_*n*_) and time *t*_*n*_, then we have λ_*n*_ = λ(*x*_*n*_, *y*_*n*_, *t*_*n*_). λ(*x*, *y*, *t*) describes the spatial-temporal distribution of background radiation level. The probability of measuring *d*_*n*_ from Poisson distribution *P*_*n*_ can be then calculated using the probability mass function:

$$\begin{array}{c} P_{n}\left(d_{n}\right)=\frac{\lambda {\left(x_{n},y_{n},t_{n}\right)}^{d_{n}}e^{-\lambda (x_{n},y_{n},t_{n})}}{d_{n}!} \end{array}$$ (1)

Since the sensor measurements {*d*_*n*_: *n* = 1, …, *n*} are all made at non-overlapping times and locations, these measurements are statistically independent. The likelihood of measuring the whole dataset D given the background radiation level λ(*x*, *y*, *t*) is a multiplicity of all the individual probability mass functions:

$$\begin{array}{c} P\left(D\right)=\prod_{n=1}^{N}P_{n}\left(d_{n}\right)=\prod_{n=1}^{N}\frac{\lambda {\left(x_{n},y_{n},t_{n}\right)}^{d_{n}}e^{-\lambda (x_{n},y_{n},t_{n})}}{d_{n}!} \end{array}$$ (2)

Calculating the natural logarithm on both sides of Eq 2, we obtain the log-likelihood of parameter λ(*x*, *y*, *t*) with respect to dataset D:

$$\begin{array}{c} l\left(\lambda \left(x,y,t\right);D\right)=\sum_{n=1}^{N}\{d_{n}log\left(\lambda \left(x_{n},y_{n},t_{n}\right)\right)-\lambda \left(x_{n},y_{n},t_{n}\right)-log\left(d_{n}!\right)\} \end{array}$$ (3)

The optimization problem is to find a background radiation distribution λ(*x*, *y*, *t*) that maximizes the log-likelihood l(λ(x,y,t);D):

$$\begin{array}{c} \hat{\lambda (x,y,t)}=\underset{\lambda (x,y,t)>0}{argmax}\sum_{n=1}^{N}\{d_{n}log\left(\lambda \left(x_{n},y_{n},t_{n}\right)\right)-\lambda \left(x_{n},y_{n},t_{n}\right)-log\left(d_{n}!\right)\} \end{array}$$ (4)

Two assumptions about the background radiation level λ(*x*, *y*, *t*) are made to separate its spatial and temporal part, and to discretize the optimization problem in both space and time. These two assumptions are made based on the properties of background radiation:

1. Spatial distribution assumption: At a given time, the spatial distribution of background radiation level is uniform over a sufficiently small area, for example 4.20*m* × 5.80*m* (length × width).
2. Temporal fluctuation assumption: The temporal fluctuation of background radiation level is the same for our experimental area: 462.0*m* × 301.6*m* (length × width).

The first assumption is based on the fact that the majority of background radiation comes from NORMs in air, soil, and building materials. In fields or cities, these NORMs are always uniformly distributed in regions that are small enough. The smaller a region is, the more uniformly those NORMs will distribute, and thus the more uniform background a region will have. However, in order to get enough measurements in each small region for statistical inference, the region size should not be too small. In this paper, data were taken along sidewalks that were at least 2 meters to buildings and featured a slow change of soil and building materials. For this scenario, these regions are chosen to be 4.20*m* × 5.80*m*. The second assumption is based on the observation that the major reason for background radiation temporal fluctuation is weather, especially precipitation [12, 16]. For an area as large as 462.0*m* × 301.6*m*, the weather condition will be the same, and thus the fluctuation behavior of background radiation is the same.

According to the second assumption, the λ(*x*, *y*, *t*) for our experimental area is separable between position (*x*, *y*) and time *t*. We further assume λ(*x*, *y*, *t*) can be separated in an additive way in our whole experimental area:

$$\begin{array}{c} \lambda \left(x,y,t\right)=\lambda_{1}\left(x,y\right)+\lambda_{2}\left(t\right) \end{array}$$ (5)

The validity of this separation will be justified in Results and Discussion section. λ_1_(*x*, *y*) is the spatial component of Poisson parameter, which represents the background radiation’s spatial distribution corresponding to building materials and soil components. These radioactive sources do not change with time, but have different distributions at different positions. λ_2_(*t*) is the temporal component of Poisson parameter, which stands for background radiation’s temporal fluctuation (i.e. caused by precipitation). According to the second assumption, such fluctuation is independent of positions in the experimental area. λ_1_(*x*, *y*) and λ_2_(*t*) are independent components in Poisson parameters controlling the spatial distribution and temporal fluctuation. Bringing Eq 5 into Eq 4, we obtain the optimization problem with time and space decoupled:

$$\begin{array}{c} {\hat{\lambda }}_{1},{\hat{\lambda }}_{2}=\underset{\lambda_{1}\left(x,y\right)+\lambda_{2}\left(t\right)>0}{argmax}\sum_{n=1}^{N}\{d_{n}log\left(\lambda_{1}\left(x_{n},y_{n}\right)+\lambda_{2}\left(t_{n}\right)\right)-\lambda_{1}\left(x_{n},y_{n}\right)-\lambda_{2}\left(t_{n}\right)\} \end{array}$$ (6)

**Fig 2:**
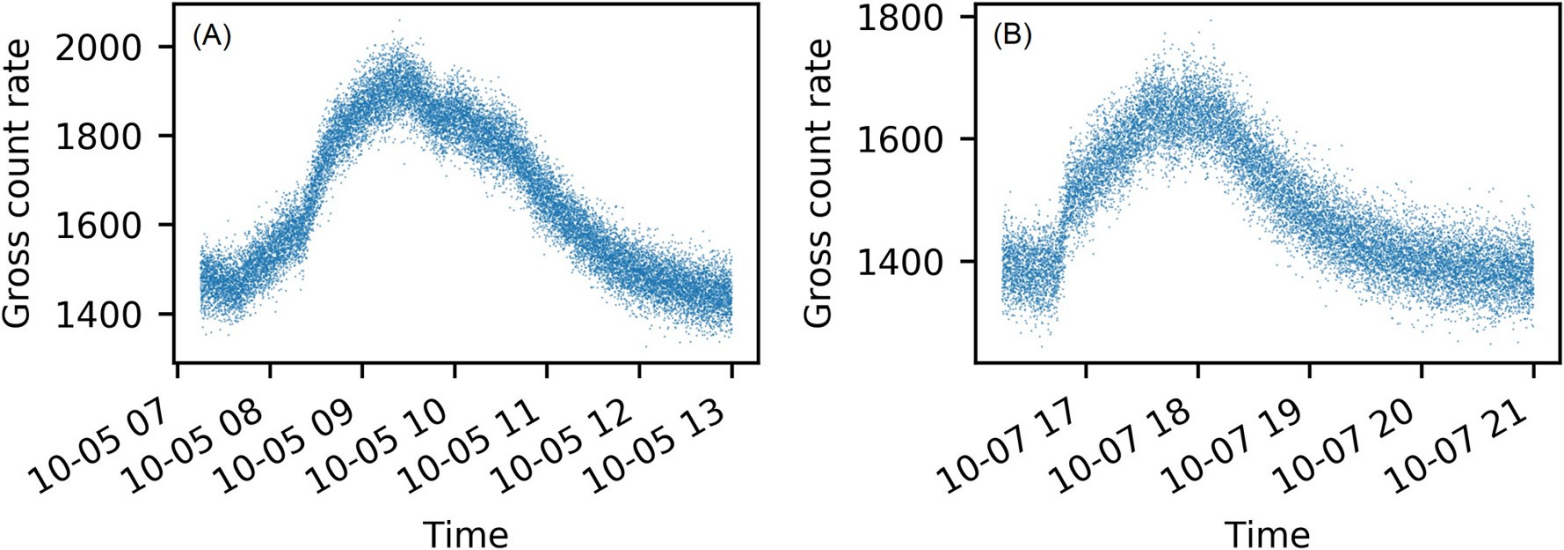
Two examples of precipitation-induced background fluctuation peaks measured by Det-1. Each dot is a one second background measurement. Panel (A) shows the precipitation-induced background peak on Oct-05 with a peak duration of 4 hours. Panel (B) shows the precipitation-induced background peak on Oct-07 with a peak duration of 3.5 hours.

In order to further simplify the optimization problem, we discretize the model in both space and time. Based on the the first assumption, the experimental area is discretized into 4.20*m* × 5.80*m* blocks with *B*_*ij*_ denoting the block that ranks i’th in the longitude direction and j’th in the latitude direction. In each of these blocks, the background radiation is assumed to be uniformly distributed and independent of surrounding blocks. We also discretize the time *t* into a series of time grids {*T*_1_, *T*_2_, …, *T*_*k*_, …}. If the time grids are too sparse, we will not be able to capture the background fluctuation peaks; on the other hand, if the time grids are too dense, we will not have enough number of measurements in each time grid to provide stable estimations. Fig 2 plots two typical precipitation-induced background fluctuation peaks in our experiment, which shows a typical peak duration of 3.5 hours. Considering this peak duration, we choose 5min as the grid size for the time grids. Under this setup, we have about 300 one-second measurements for each time grid, and we have about 40 time grids to model a temporal fluctuation peak.

With these modifications, the λ_1_(*x*, *y*) and λ_2_(*t*) can be represented as follows:

$$\begin{array}{cc} & \lambda_{1}\left(x,y\right)=\alpha_{ij},\;where\;\left(x,y\right)\in B_{ij} \end{array}$$ (7)

$$\begin{array}{cc} & \lambda_{2}\left(t\right)=\beta_{k},\;where\;t\in T_{k} \end{array}$$ (8)

Here ***α*** is a matrix storing all the spatial components of background radiation in our experimental area, and ***β*** is a vector storing all the temporal components of background radiation during our experiment. Brining Eqs 7 and 8 into Eq 6, we obtain the final object function *L* to optimize for this problem:

$$\begin{array}{c} L\left(\alpha ,\beta ;D\right)=\sum_{n=1}^{N}\sum_{i,j,k}\{d_{n}log\left(\alpha_{ij}+\beta_{k}\right)-\alpha_{ij}-\beta_{k}\}1_{\left\{\left(x_{n},y_{n}\right)\in B_{ij}\;and\;t_{n}\in T_{k}\right\}}) \end{array}$$ (9)

And the optimization problem can be written as follows:

$$\begin{array}{cc} \underset{\alpha ,\beta }{max}\; & L(\alpha ,\beta ;D) \end{array}$$ (10)

$$\begin{array}{cc} \text{subject}\;\text{to} & \alpha_{ij}+\beta_{k}>0,\forall i,j,k \end{array}$$ (11)

$$\begin{array}{cc} \text{and} & \beta_{0}=0 \end{array}$$ (12)

Because the objective function *L* is concave and second-order differentiable, we implemented the Newton–Raphson method [17] to solve this optimization task.

### Experimental validation of the temporal fluctuation assumption

Two stationary detectors (named Det-1 and Det-2) were set up on campus to record the temporal fluctuation of background radiation. Their measurements were used to validate the temporal fluctuation assumption in the BR-MLE algorithm.

**Fig 3:**
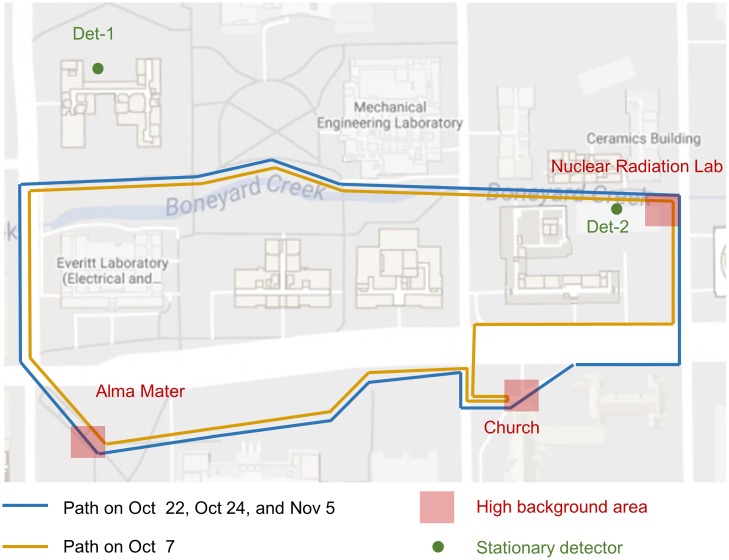
The setup of detectors in the experimental area. This map is roughly 462.0 meters long and 301.6 meters wide. It has three high background areas: the Alma Mater, the Church, and the Nuclear Radiation Lab. Two stationary detectors, the Det-1 and the Det-2, were setup at two different locations with a distance of 338 meters. In the experiment of background radiation estimation, the mobile sensor network scanned the experimental area in blue or yellow paths on different days.

These two stationary detectors are both from the Saint-Gobain Crystals with the same model number 2X4H16/2SS. They are sodium iodide scintillation detectors with crystal size 2 × 4 × 16 inch. The high voltages of these two detectors were calibrated with each other using a ^137^Cs source so that the ^137^Cs’s full energy peaks measured by these two detectors were at the same energy channel. After the energy calibration, these two stationary detectors have the same gamma-ray detection range which is between 30keV to 3MeV. This range is the same as the D3S detectors in the mobile sensor network. Both of the two stationary detectors are configured to report radiation measurements every second with detection time interval of one second. This study focuses on the gross count rate of background radiation, thus the gross count responses of these two detectors were calibrated again using a ^137^Cs source. After this calibration, these two stationary detectors were setup at two locations shown in Fig 3. Det-1 is on the roof of a one-story building at latitude 40.112046° and longitude -88.228347°, while Det-2 is on the roof of another one-story building at latitude 40.111289° and longitude -88.224502°. The distance between these two stationary detectors is 338 meters. Near the Det-1, we also setup a weather station to record weather conditions in every ten minutes. A total of 23 days’ background radiation and weather measurements were recorded.

### Experiment of background radiation estimation

In this experiment, the background radiation of an experimental area was measured by a one-node mobile sensor network on four different days. Based on those measurements, the spatial distribution and temporal fluctuation of background radiation were then estimated using the BR-MLE algorithm. In order to test the system’s performance under fluctuating background radiation, both the experimental area and the experimental days were specially chosen such that there were substantial background fluctuations in both space and time. On each day of the experiment, an operator holding the detection node walked in pre-defined paths for several laps with a usual walking speed. Throughout the experiment, the detection node was held in the same fashion and the distances from body were maintained the same. The geopositions, time stamps, and radiation count rates were automatically recorded once per second.

The average background radiation of the experimental area was 46 cps (counts per second) measured by the D3S detectors. Several places in the experimental area (denoted by red rectangles in Fig 3) have elevated background radiation due to their building materials. These places’ background radiation levels were between 65 cps and 95 cps measured by the D3S detectors. The scanning paths were carefully designed such that they came across those naturally high background regions. The first day’s path was slightly different from the other three days’ paths because of road construction. During this experiment, rain happened on each of the four days and caused the background radiation to fluctuate temporally.

## Results and discussion

### Experimental validation of the temporal fluctuation assumption

In the BR-MLE algorithm, we assumed (second assumption) that the temporal fluctuation of background radiation is the same for our experimental area. To validate this, we setup two stationary detectors (named Det-1 and Det-2) at two locations in our experimental area, and measured the background radiation for 23 days.

**Fig 4:**
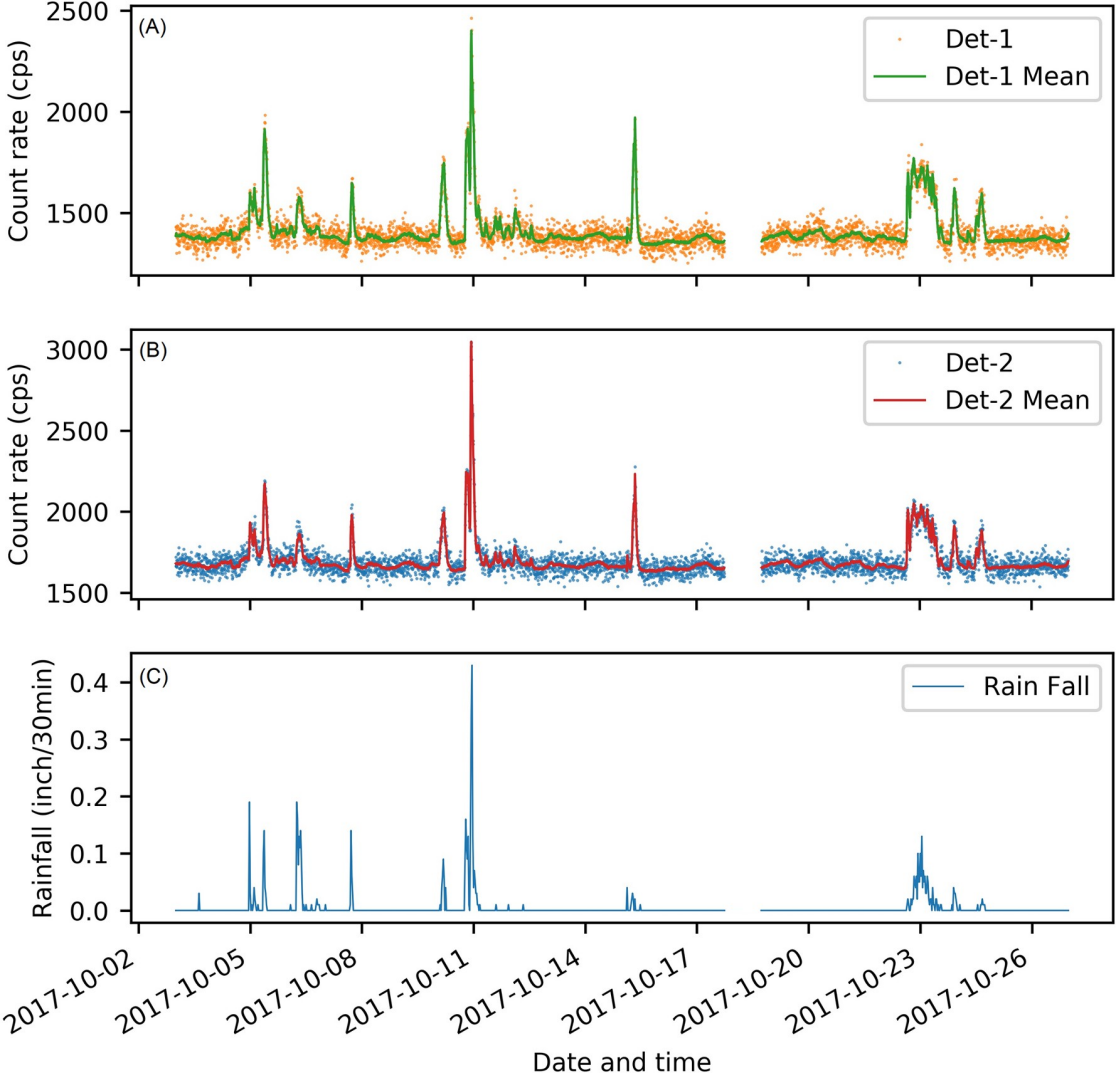
Background radiation and weather measurements for 23 days. (A) Temporal fluctuations of background radiation measured by the Det-1 (S1 Table). (B) Temporal fluctuations of background radiation measured by the Det-2 (S2 Table). (C) Precipitation levels measured by the weather station (S3 Table). In the first two plots, dots are the one-second background measurements, and lines are the mean value of these measurements in a 5-min time interval.

Fig 4 shows the 23 days’ radiation measurements from Det-1 and Det-2, and the precipitation measurements from the weather station near Det-1. For both Det-1 and Det-2, all the prominent peaks of background radiation fluctuation were corresponding to raining events. This validates that rainfall is the major cause for temporal fluctuation of background radiation. Fig 4 also indicates that the baseline levels of background radiation were different between Det-1 and Det-2. Without raining, the background radiation at Det-1 was around 1388 cps, while the background radiation at Det-2 was around 1679 cps. Though with different background radiation, Det-1 and Det-2 fluctuated in the same manner during raining events.

**Fig 5:**
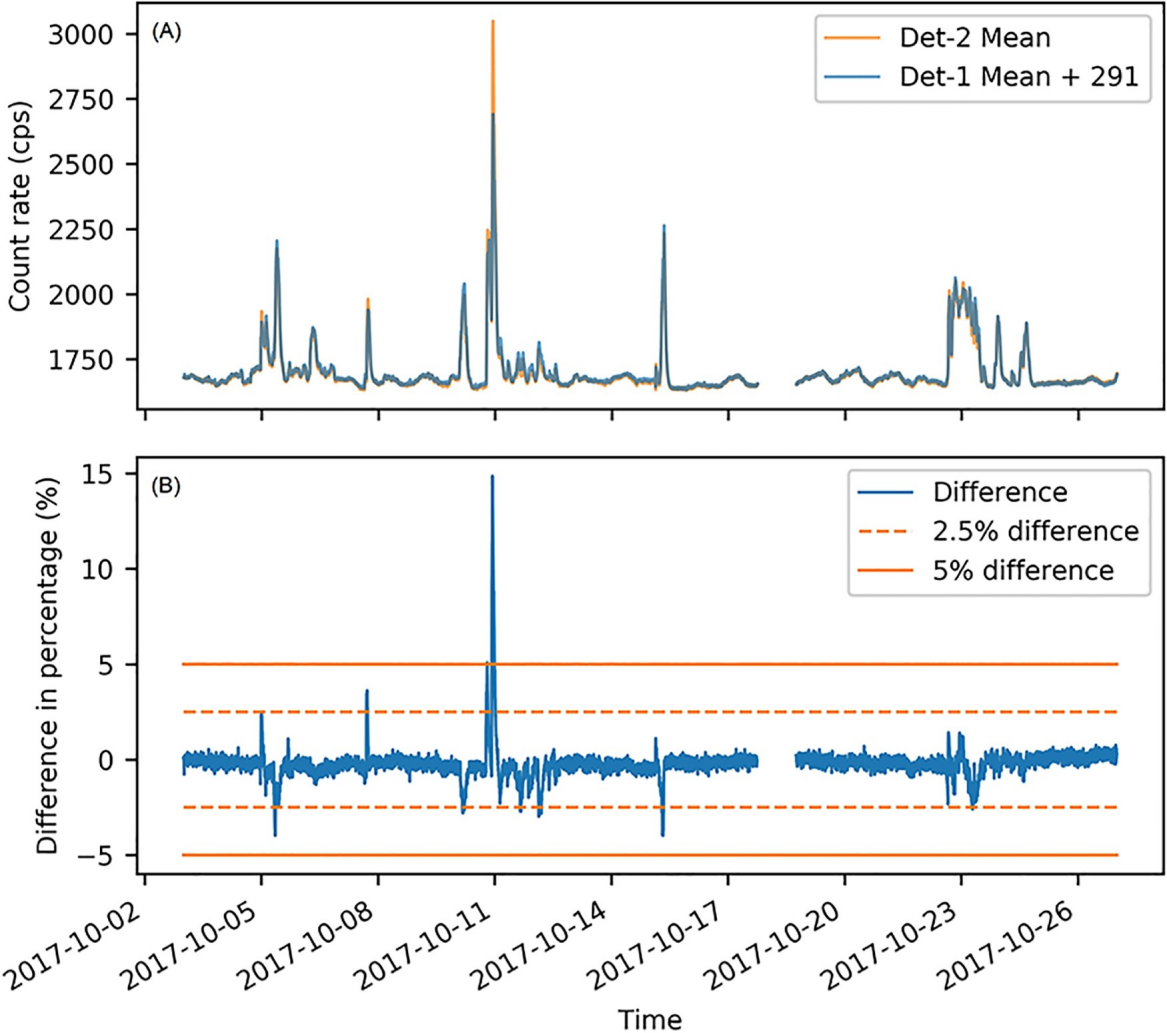
Comparison of background measurements between Det-1 and Det-2. (A) The upper panel shows the overlapped background radiation measurements from Det-1 and Det-2. The baseline difference of background radiation between Det-1 and Det-2 was 291 cps. After adding 291 cps, the measurements from Det-1 were almost the same as the measurements from Det-2 except for the raining event around 2017-10-10 23:00. (B) The lower panel shows the difference in percentage between Det-1 (denote its measurements by *d*1) and Det-2 (denote its measurements by *d*2). The difference is calculated as: difference = (*d*2 − (*d*1 + 291))/(*d*1 + 291). During the 23 days, the differences between Det-1 and Det-2 were within 2.5 percent for most of the time. The only time that this difference exceeded 5 percent happened during the rain at midnight of 2017-10-11.

Fig 5 compares the background radiation between Det-1 and Det-2. After adding 291 cps to Det-1, the measurements from Det-1 were almost the same as Det-2, except for the time period between 2017-10-10 22:20 and 2017-10-11 00:00. During this time period, the differences between Det-1 and Det-2 reached 15 percent, while the other time’s differences were lower than 5 percent as shown in the lower plot of Fig 5. This significant abnormal difference between Det-1 and Det-2 may be caused by the strong precipitation from 2017-10-10 22:20 to 2017-10-11 00:00. During this 100 minutes, the precipitation rate reached 0.43 inch/30min, which was already two-fold of the second highest precipitation rate during the 23 days (shown by the bottom plot of Fig 4). This strong rainfall rate may already exceed the water drainage capacity of the roof placing Det-2 and cause water to accumulate excessively. Because rain water dissolves the major contributors that elevate background radiation [18], the excessive accumulation of rain water can finally lead to a higher peak in background temporal fluctuation around Det-2 than Det-1. In other raining events during our experiment, the rainfall rate may not be high enough to cause the excessive accumulation of water. Because of this abnormal difference between Det-1 and Det-2, the measurements between 2017-10-10 22:20 and 2017-10-11 00:00 were treated as outliers and excluded in the following linear regression analysis. In the remaining dataset, the precipitation rates were no higher than 0.2 inch/30min.

**Fig 6:**
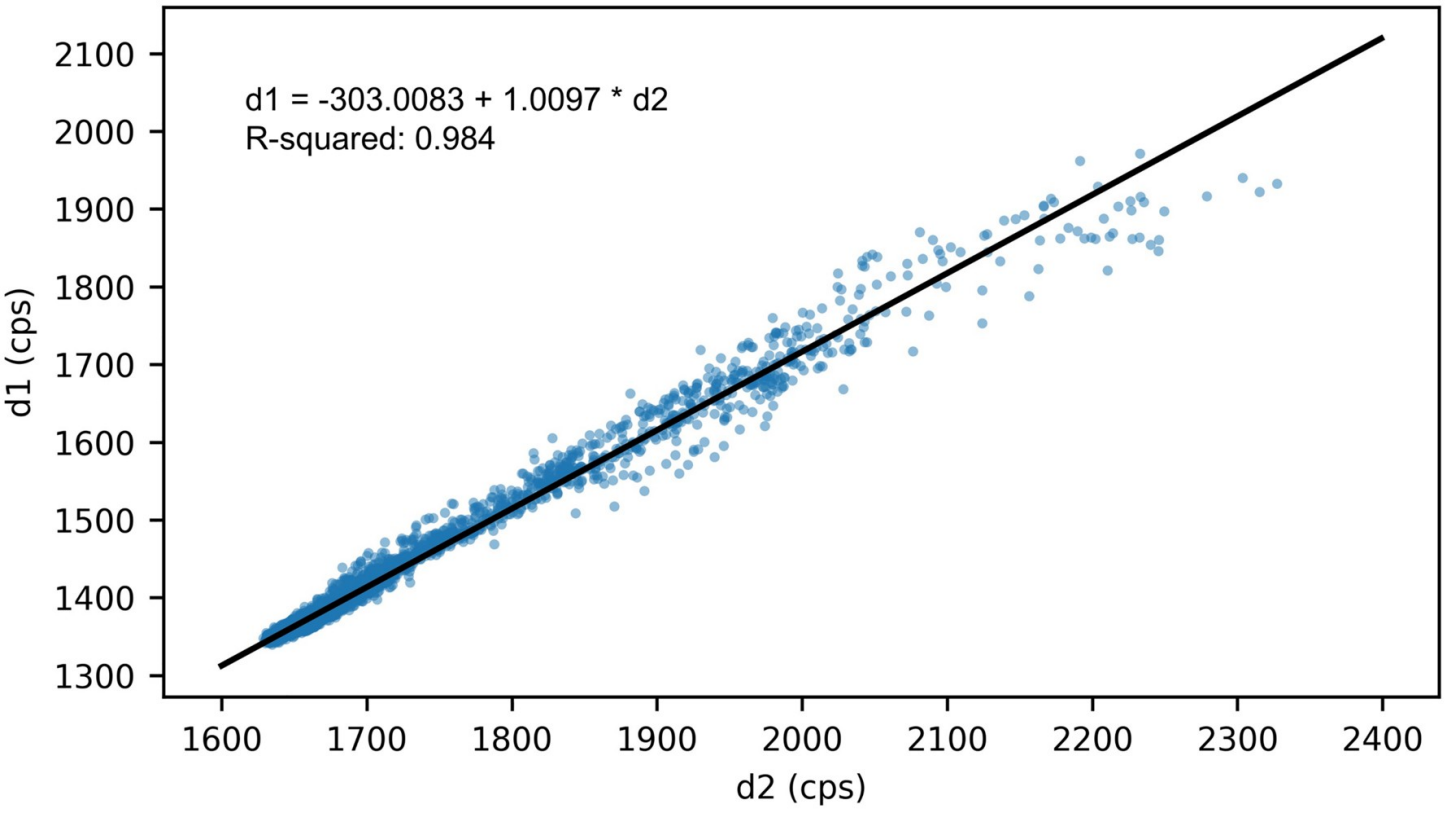
Results of linear regression between Det-1’s measurements (denoted by *d*1) and Det-2’s measurements (denoted by *d*2). The slope of the fitted line is very close to one. This indicates that the amplitudes of the background radiation temporal fluctuation are the same for Det-1 and Det-2. The interception of the fitted line indicates that the baseline difference of background radiation between Det-1 and Det-2 is 303 cps.

To further validate the temporal fluctuation assumption, we used a linear regression model to fit Det-2’s measurements (named as *d*2) to Det-1’s measurements (named as *d*1). As shown in Fig 6, the linear regression achieves a 0.984 R-squared value which indicates the relationship between *d*1 and *d*2 can be properly modeled by the fitted line. The interception of the fitted line, -303 cps, shows the baseline difference of background radiation between Det-1 and Det-2. This difference is close to our previous measured value: -291 cps. The slope, 1.0097, is very close to 1. This indicates *d*1 and *d*2 have the same fluctuation amplitude, though their background baselines are different. Considering the Det-1 and the Det-2 are relatively far away in our experimental area, this result is representative for the whole experimental area. This validates the temporal fluctuation assumption that the background radiation’s temporal fluctuation is the same for our experimental area. This also validates the Eq 5, which assumes that the background radiation distribution λ(*x*, *y*, *t*) can be split into spatial and temporal components in an additive way.

### Experiment of background radiation estimation

A total of six hours’ radiation measurements were collected by the mobile sensor network during this experiment (S4 Table). Based on these measurements, the spatial distribution and temporal fluctuation of background radiation were estimated using the BR-MLE algorithm.

**Fig 7:**
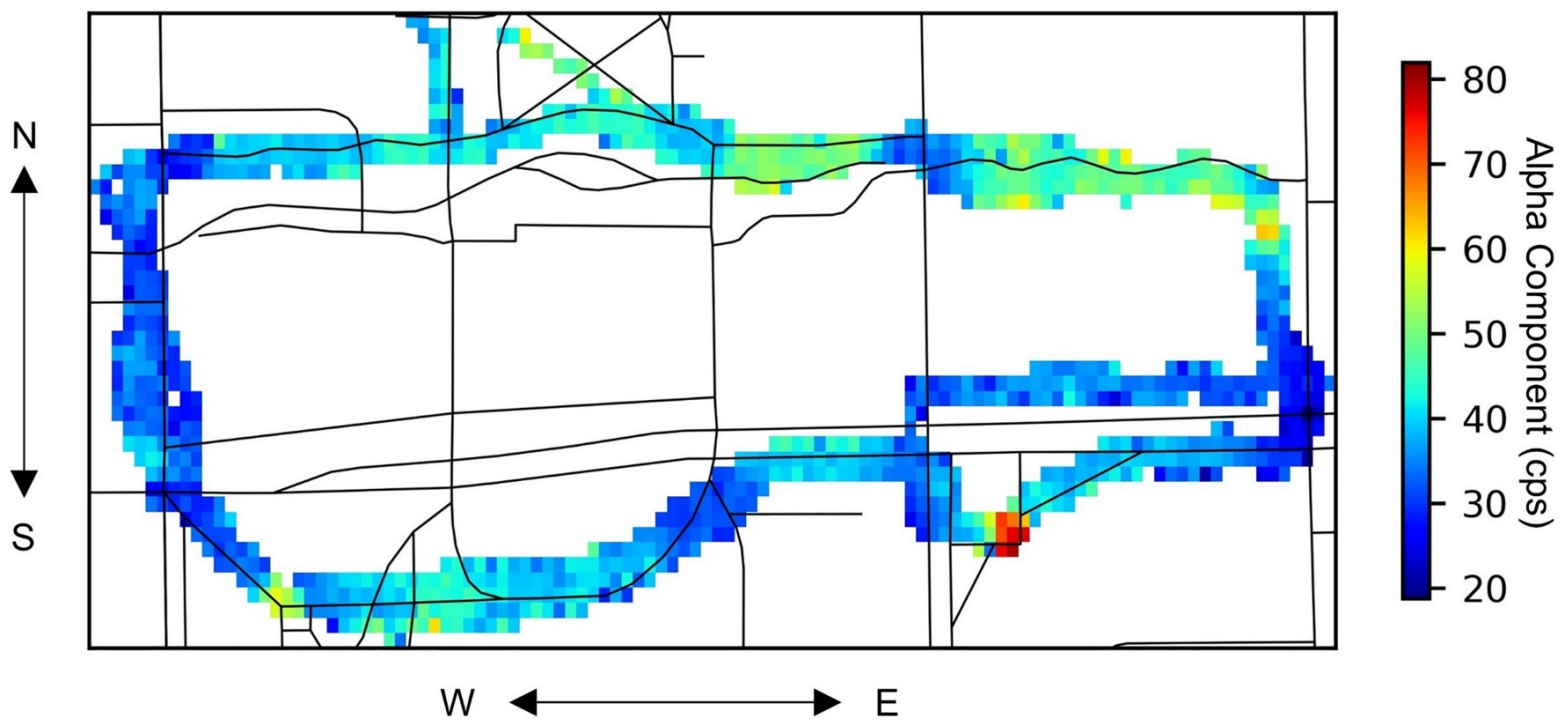
Estimated spatial distribution of background radiation from the BR-MLE algorithm. This figure plots the heat map of *α* calculated by the BR-MLE algorithm, which illustrates the background radiation distribution in the southern part of the map shown in Fig 3. This plotted area has size 462.0*m* × 243.6*m* with 110 × 42 grids. Each grid size is 4.20*m* × 5.80*m*.

Fig 7 shows the heat map of ***α*** calculated by the BR-MLE algorithm. According to Eq 7, the ***α*** illustrates the experimental area’s background radiation distribution. The mean value of ***α*** for the experimental area was 39 cps. Areas around the previously-identified high background regions in Fig 3 had higher radiation count rates, as expected. The ***α*** values of the church area, the nuclear radiation laboratory, and the Alma Mater area were 80 cps, 58 cps, and 63 cps respectively.

**Fig 8:**
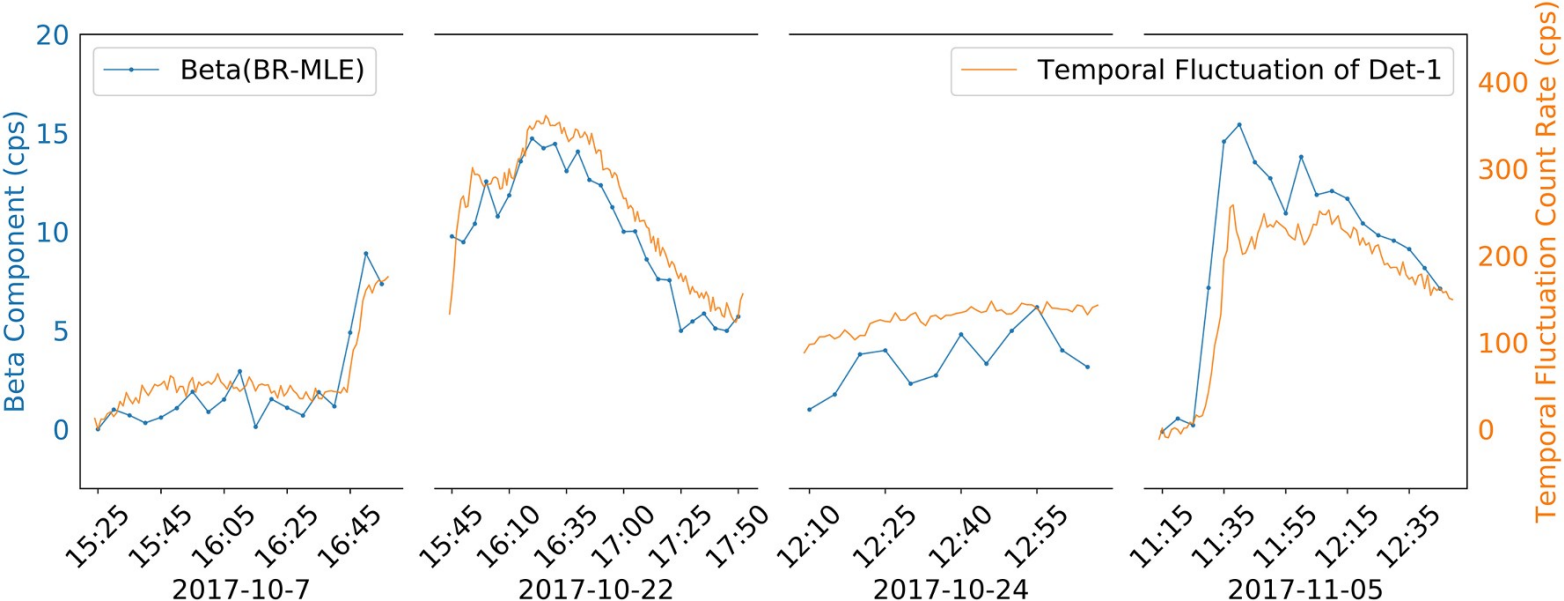
Temporal fluctuation components (*β*) of background radiation for the four days’ experiment. The blue dotted-lines show the estimated background fluctuation components (***β***) from the BR-MLE algorithm, and they use the y axis on the left hand side. The yellow dotted-lines show the measured background fluctuations from the Det-1 detector, and they use the y-axis on the right hand side. Because the D3S detector’s crystal size is much smaller than the Det-1 detector, the gross count rate from the D3S detector is much lower than the Det-1 detector, and thus the blue line and the yellow line have different y-axis scales. The temporal fluctuation estimations from the BR-MLE algorithm matched the background fluctuations measured by the Det-1 detector.

Fig 8 compares the estimated temporal fluctuation from the BR-MLE algorithm and the measured temporal fluctuation from the Det-1 detector. On 2017-10-07, 2017-10-22, and 2017-11-05, the background radiation changed significantly; on 2017-10-24, the background radiation had a slowly increasing trend. In the four days’ experiment, the Det-1 detector measured a maximum background radiation of 1706 cps during the rain of 2017-10-22, which was 350 cps higher than the background radiation without rain (1356 cps). This indicates the necessity to take temporal fluctuation into consideration when modeling an area’s background radiation. As shown in Fig 8, the temporal fluctuation estimations from the BR-MLE algorithm were consistent with the background fluctuations measured by the Det-1 detector.

## Conclusion

In this study, we demonstrated the usage of mobile sensor networks to monitor an area’s background radiation. A mobile sensor network was built to monitor the campus’ background radiation of the University of Illinois. A background radiation estimation algorithm, the BR-MLE algorithm, was developed to model the background radiation. Experimental results show that this background radiation monitoring system correctly reconstructed the spatial distribution and temporal fluctuation of background radiation. High background areas were correctly identified, and the temporal fluctuations estimated by the BR-MLE algorithm were consistent with the direct observations measured by the stationary detectors.

## Supporting information

S1 Table Background radiation measurements from the stationary detector Det-1. The *local_time* column is in the time zone of Central Time (CT), United States. Radiation measurements are in the unit of cps (counts per second).
(CSV)

S2 Table Background radiation measurements from the stationary detector Det-2. The *local_time* column is in the time zone of Central Time (CT), United States. Radiation measurements are in the unit of cps (counts per second).
(CSV)

S3 Table Precipitation measurements from the weather stationary near Det-1. The *datetime* column is in the time zone of Central Time (CT), United States. Precipitation measurements are in the unit of inch per half hour.
(CSV)

S4 Table Background radiation measurements from the mobile sensor network. The *time* column is in the unit of epoch. Radiation measurements are in the unit of cps (counts per second).
(CSV)
